# Machine learning combined with radiomics and deep learning features extracted from CT images: a novel AI model to distinguish benign from malignant ovarian tumors

**DOI:** 10.1186/s13244-023-01412-x

**Published:** 2023-04-24

**Authors:** Ya-Ting Jan, Pei-Shan Tsai, Wen-Hui Huang, Ling-Ying Chou, Shih-Chieh Huang, Jing-Zhe Wang, Pei-Hsuan Lu, Dao-Chen Lin, Chun-Sheng Yen, Ju-Ping Teng, Greta S. P. Mok, Cheng-Ting Shih, Tung-Hsin Wu

**Affiliations:** 1 Department of Biomedical Imaging and Radiological Sciences, National Yang Ming Chiao Tung University, Taipei, 112 Taiwan; 2 Department of Radiology, MacKay Memorial Hospital, Taipei, Taiwan; 3 Department of Medicine, MacKay Medical College, New Taipei City, Taiwan; 4 MacKay Junior College of Medicine, Nursing and Management, New Taipei City, Taiwan; 5 Division of Endocrine and Metabolism, Department of Medicine, Taipei Veterans General Hospital, Taipei, Taiwan; 6 Department of Radiology, Taipei Veterans General Hospital, Taipei, Taiwan; 7 School of Medicine, National Yang Ming Chiao Tung University, Taipei, Taiwan; 8 Biomedical Imaging Laboratory (BIG), Department of Electrical and Computer Engineering, Faculty of Science and Technology, University of Macau, Macau, China; 9 Department of Biomedical Imaging and Radiological Science, China Medical University, Taichung, 404 Taiwan

**Keywords:** Ovarian tumor, Radiomics, Deep learning, Machine learning, Computed tomography

## Abstract

**Background:**

To develop an artificial intelligence (AI) model with radiomics and deep learning (DL) features extracted from CT images to distinguish benign from malignant ovarian tumors.

**Methods:**

We enrolled 149 patients with pathologically confirmed ovarian tumors. A total of 185 tumors were included and divided into training and testing sets in a 7:3 ratio. All tumors were manually segmented from preoperative contrast-enhanced CT images. CT image features were extracted using radiomics and DL. Five models with different combinations of feature sets were built. Benign and malignant tumors were classified using machine learning (ML) classifiers. The model performance was compared with five radiologists on the testing set.

**Results:**

Among the five models, the best performing model is the ensemble model with a combination of radiomics, DL, and clinical feature sets. The model achieved an accuracy of 82%, specificity of 89% and sensitivity of 68%. Compared with junior radiologists averaged results, the model had a higher accuracy (82% vs 66%) and specificity (89% vs 65%) with comparable sensitivity (68% vs 67%). With the assistance of the model, the junior radiologists achieved a higher average accuracy (81% vs 66%), specificity (80% vs 65%), and sensitivity (82% vs 67%), approaching to the performance of senior radiologists.

**Conclusions:**

We developed a CT-based AI model that can differentiate benign and malignant ovarian tumors with high accuracy and specificity. This model significantly improved the performance of less-experienced radiologists in ovarian tumor assessment, and may potentially guide gynecologists to provide better therapeutic strategies for these patients.

**Supplementary Information:**

The online version contains supplementary material available at 10.1186/s13244-023-01412-x.

## Key points

1. CT-based radiomics and deep learning features could differentiate ovarian tumors.
2. Radiomics, deep learning features, and clinical data provided complementary tumor information.
3. The ensemble model improved the radiologists’ performance in assessing ovarian tumors.

**Supplementary Information:**

The online version contains supplementary material available at 10.1186/s13244-023-01412-x.

## Background

Ovarian cancer is the leading cause of gynecological cancer related deaths [1], and a misdiagnosis may delay the treatment and worsen the prognosis. Expedited referral of patients with ovarian cancer to a gynecologic oncologist for complete surgical staging and optimal cytoreduction correlates with better survival rates [2]. In contrast, patients with benign ovarian tumor only need conservative treatment or laparoscopic cystectomy [3]. Therefore, accurate distinction between benign and malignant ovarian tumors is of paramount importance in guiding treatment and it remains a great challenge in clinical practice.

Currently, distinction between benign and malignant ovarian tumors is largely based on imaging appearance [4–6]. Ultrasound is typically the first-line screening imaging tool. Due to the excellent spatial resolution and wide availability, computed tomography (CT) is often ordered for further tumor characterization. However, a definitive differentiation between benign and malignant ovarian tumors by CT remains challenging, especially in excluding the possibility of malignancy in multiseptated cystic tumors. Given that benign ovarian tumors greatly outnumber malignant ones, it is not uncommon that patients with tumor of indeterminate image features undergo surgery and the tumors are later proven to be benign. It is estimated that approximately 28% of oophorectomies performed are of benign tumors [7]. These unnecessary surgeries represent a huge clinical concern with long-term consequences of decreased fertility and premature menopause [8, 9]. Therefore, a noninvasive method that can accurately distinguish benign from malignant ovarian tumors to prevent delayed treatment in malignant cases and save patients with benign tumors from unnecessary surgery is of significant clinical impact.

Artificial intelligence (AI) has been shown to improve the performance of tumor detection, tumor classification, and treatment monitoring in cancer imaging [10–13]. In contrast with subjective radiological imaging evaluation by humans, image feature extraction using radiomics or deep learning (DL) can provide quantified image information undetectable by human eyes and has shown promising results in tumor analysis [14–25]. Several recent studies used radiomics on CT images and applied machine learning (ML) classifiers to differentiate ovarian tumors [26–28]. However, there is limited research on applying DL to differentiate ovarian tumor using CT images. Christiansen et al. [29] and Wang et al. [30] applied DL for ovarian tumor differentiation using ultrasound and magnetic resonance imaging (MRI) respectively. In addition to studies that directly applied DL networks for ovarian tumor differentiation, there were few studies using DL networks for feature extraction from CT images to predict ovarian cancer recurrence or classify pulmonary nodule subtypes [24, 25]. To our best knowledge, the performance of applying ML based on combined radiomics and DL features extracted from CT images on differentiating ovarian tumors remains unknown.

In this study, we aimed to develop a CT-based AI model with feature extraction using radiomics and DL to distinguish benign from malignant ovarian tumors. We applied classifiers with radiomics and DL features extracted from CT images to classify benign and malignant ovarian tumors. The performance of various combinations of classifiers and feature sets were compared with radiologists on the classification task using pathologic diagnosis as the gold standard. Moreover, the performance improvement of radiologists with assistance of the optimal model was also assessed.

## Methods

### Study population

**Fig. 1:**
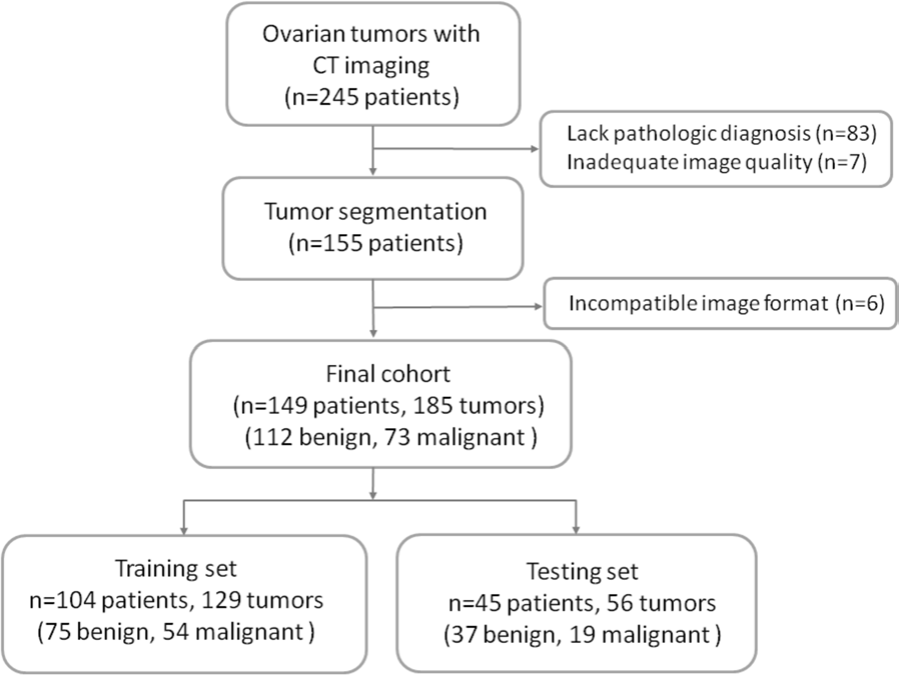
Flowchart of patient selection

In this institutional review board-approved study, we retrospectively collected 245 consecutive patients with suspected ovarian tumors from the MacKay Memorial Hospital between July 2018 and December 2019. Patients meeting the following criteria were included: (1) pathologically confirmed ovarian tumor resected by surgery, (2) contrast-enhanced CT scan performed prior to surgery, (3) clear CT images without artifacts and fit for analysis. The final cohort consisted of 149 patients with 185 ovarian tumors (Fig. 1).

**Fig. 2:**
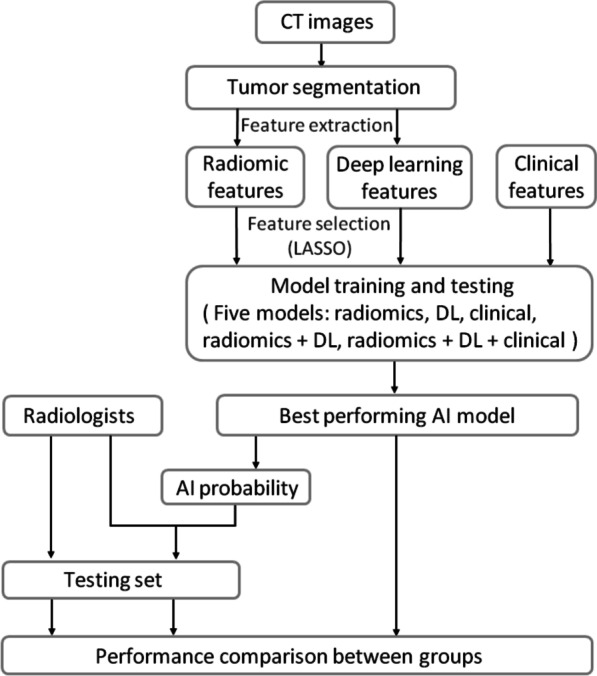
Workflow of study design

The data were divided into training and testing sets in a 7:3 ratio. The training set was used to develop five models with different combinations of feature sets: radiomics model, DL model, clinical model, combined radiomics and DL model, and ensemble model (combined radiomics, DL, and clinical feature sets). The models were then tested on the unseen testing set. Figure 2 illustrates the flowchart of study design.

### Image acquisition and segmentation

CT examinations were performed on 4 different multidetector CT scanners: Siemens Somatom Definition Flash, Siemens Somatom Definition AS, Toshiba Aquilion ONE (TSX-301C), Toshiba Aquilion PRIME (TSX-303A). The scanning parameters were as follows: tube voltage, 120 kVp; tube current, 200–230 mA; gantry rotation time, 0.5 s; beam pitch, 1.0; reconstruction thickness, 2 mm; reconstruction interval, 1.5 mm. Contrast medium (Iodine concentration: 300 mg/mL) 80–100 mL was injected using a mechanical injector at a rate of 2.5–3.5 mL/sec. The time delay from contrast agent injection to image acquisition was 70 s.

The preoperative contrast-enhanced CT images were collected from the PACS. Tumors were manually segmented by an experienced radiologist using 3D slicer (IEEE Cat No. 04EX821). The boundary of the whole tumor was manually defined on each axial CT slice.

### Feature extraction, selection, and tumor classification

After resolution and intensity normalization, radiomics features were extracted from the tumor images. A total of 129 radiomics features were extracted from each tumor, including 12 histogram features, 9 gray-level co-occurrence matrix (GLCM) features, 96 wavelet features, and 12 Laplacian of Gaussian (LoG) features (Additional file 1: Table S1).

**Fig. 3:**
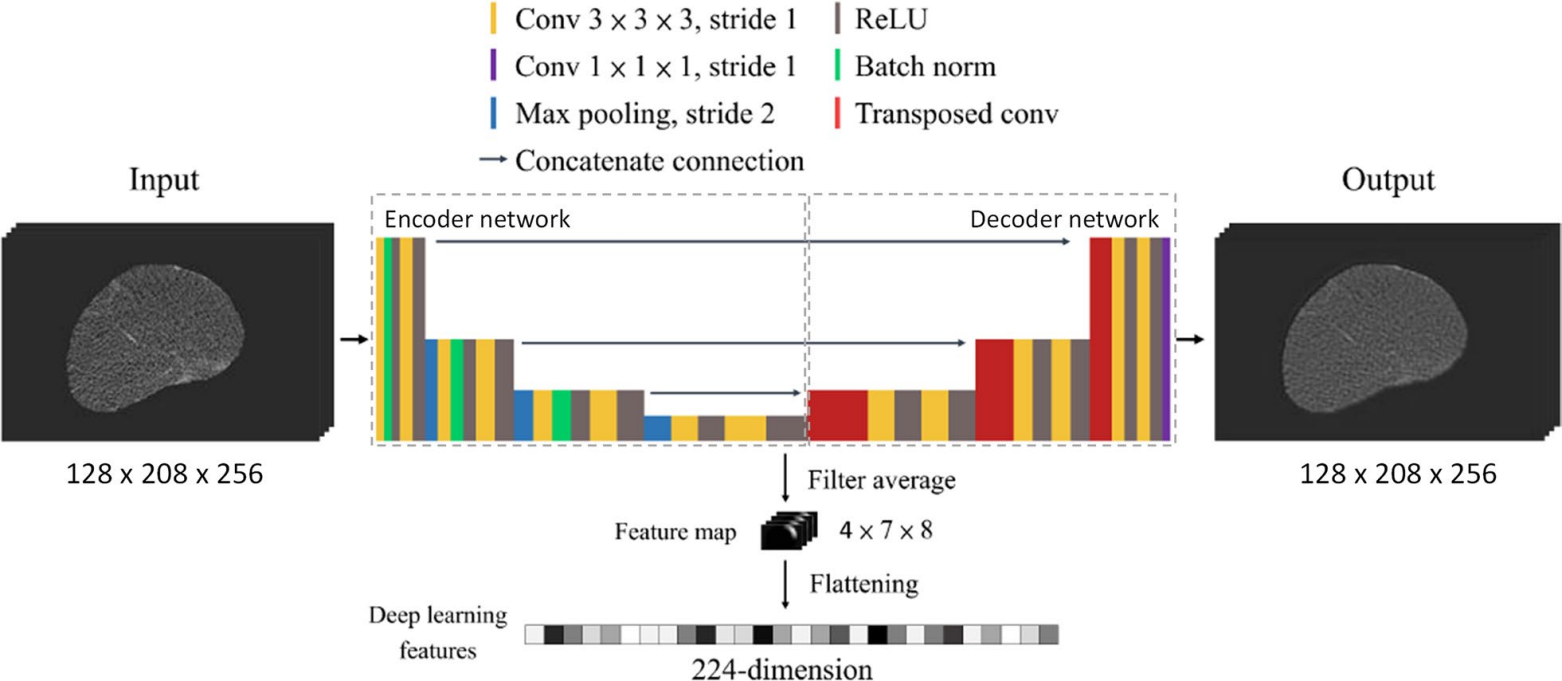
The architecture of the 3D U-net used for DL feature extraction. The architecture includes an encoder network and a decoder network. The encoder extracts tumor characteristics referred to as DL features, and the decoder uses the DL features to reconstruct original tumor image. The segmented tumor images were input into the network. The output of the last convolutional layer in the encoder network was extracted as a 224-dimensional DL feature

In addition to the radiomics, a 3D U-Net convolutional neural network (CNN) was applied as a feature extractor. Figure 3 illustrates the architecture of the U-Net applied in this study, which consists of an encoder and a decoder. The basic idea of the use of the U-net as a feature extractor is that the features extracted by the encoder from an input tumor image could represent the tumor if the image reconstructed by the decoder using the features is similar to the input image [31–34]. In this study, the U-net was trained and validated respectively by 90% and 10% of the training set using Adam optimizer with a loss function of half mean squared error. A batch size of 1 was used due to the limited memory size of the applied graphic card. The learning rate and the number of epochs for the training were adjusted based on the averaged root mean squared error (RMSE) between the input and reconstructed images to ensure the images reconstructed by the decoder were as much as similar to the input images. By inputting the tumor images to the trained U-net, the features output by the last activation layer of the encoder were adopted as DL features of the tumor. For each tumor, 224 DL features were extracted.

Using the radiomics and U-net, 353 features were extracted from each tumor. However, the performance of classification using such a large number of features could be low due to multiple collinearity and over-fitting. We used a least absolute shrinkage and selection operator (LASSO) regression with tenfold cross-validation to eliminate irrelevant features [35]. Features with regression coefficients > 0.1 were selected for the classification.

After feature selection, benign and malignant tumors were classified using four classifiers, including K-nearest neighbor (KNN), support vector machine (SVM), logistic regression (LR), and random forest (RF), with five types of feature sets, including radiomics features, DL features, clinical features, combined radiomics and DL features, and ensemble features (all features combined). The classification result would output a probability (0–100%) of malignancy for each tumor. The performance of the classification using different combinations of classifiers and feature sets were evaluated and compared using the training data with tenfold cross-validation. In this study, feature extraction, selection, and classifier training and evaluation were implemented using MATLAB R2020a (MathWorks, Natick, MA).

### Radiologist evaluation

Based on the years of experience reading abdominal CT images, radiologists were divided into two groups, including juniors (3 radiologists, experience < 10 years) and seniors (2 radiologists, experience > 10 years). All radiologists were blinded to patients’ pathologic diagnoses. They were asked to independently interpret the CT images of the testing set and record each tumor as benign or malignant with the given information of patients’ age and CA-125 level. After one month, they were asked to interpret the images again with the assistance of the best performing model.

### Statistical analysis

In order to evaluate the performance of the AI models and radiologists, the following indices were calculated: accuracy, sensitivity, specificity, receiver operating characteristic curve (ROC), area under the ROC curve (AUC), and F1 score. Interobserver reliability was assessed by using Krippendorff’s alpha coefficient. When assessing the clinical characteristics between groups, differences in continuous variables and categorical variables were examined using the independent samples t-test and chi-squared test, respectively. *p* < 0.05 was considered significant difference. Statistical analysis was performed using SPSS version 24.0 (IBM Corporation, Armonk, NY, USA).

## Results

### Patient demographics

The final cohort consisted of 149 patients with 185 ovarian tumors, 112 benign and 73 malignant. The patients’ age ranged from 18 to 80 years old (mean 46.4 ± 12.4 years). There were 78 patients (52.3%) with elevated CA-125 and 36 patients (24.2%) with bilateral tumors. There were significant differences in age (*p* < 0.0001), tumor volume (*p* < 0.0001), and CA-125 (*p* = 0.0003) between the benign and malignant groups (Table 1). The training and testing sets were balanced in terms of all clinical variables (Additional file 1: Table S2). Tumor histological subtypes are summarized in Table 2. For classification purposes, borderline and malignant tumors were grouped into a single category and referred to as malignant.

**Table 1:** Patient and tumor characteristics for the benign and malignant groups

	Benign (*n* = 112)	Malignant (*n* = 73)	*p* value
Age (years)	42.5 ± 13.8	52.4 ± 13.4	< 0.0001
Volume (cm^3^)	405.8 ± 532.3	1095.6 ± 1385.1	< 0.0001
*CA-125*			
≤ 35 U/mL	63 (56.3%)	21 (28.8%)	0.0003
> 35 U/mL	49 (43.7%)	52 (71.2%)	
*Side*			
Unilateral	72 (64.3%)	55 (75.3%)	0.1141
Bilateral	40 (35.7%)	18 (24.7%)	

All values are expressed as the mean ± SD or number (%)

CA-125 cancer antigen 125

**Table 2:** Summary of pathological subtypes

Category	Pathological subtype	Number
Benign (*n* = 112)	Benign epithelial tumor	38
	Benign sex-cord stromal tumor	7
	Benign germ cell tumor	20
	Benign adenomatoid tumor	1
	Endometrioma	37
	Ovarian torsion	3
	Pelvic inflammatory disease	2
	Functional cyst	4
Malignant (*n* = 73)	Borderline epithelial tumor	17
	Malignant epithelial tumor	40
	Malignant sex-cord stromal tumor	6
	Malignant germ cell tumor	2
	Metastasis	8

### Feature selection and tumor classification

The details of features selected by LASSO method are described in Table 3. In the radiomics model, 4 features were selected from initial 129 radiomics features. For the DL features, the feature extraction DL model (U-net) was trained using a learning rate of 0.001 s and 25 epochs. The average RMSE between the input and reconstructed images was 25.45 ± 39.05. Four features were selected from initial 224 DL features for DL model. In the combined radiomics and DL model, 6 features were selected from the total 353 radiomics and DL features, including one radiomics feature and five DL features. The clinical model had four clinical features: age, CA-125, tumor volume, and tumor side. The ensemble model consisted of 10 features including 4 clinical features and 6 features used in the combined radiomics and DL model. The detailed model performance on training and testing sets using different classifiers, i.e., KNN, SVM, LR, and RF, can be found in Additional file 1: Tables S3–S4. Due to the overall better performance of the LR classifier compared with other classifiers on the testing set, its analysis results were presented for evaluation for the rest of the study.

**Table 3:** Radiomics and deep learning features selected by LASSO

Model	Selected features	LASSO coefficient
Radiomics (*n* = 4)	GLCM-correlation	0.45
	Wavelet-HHL-skewness	− 0.38
	Wavelet-HHH-50th percentile	0.2
	LoG-50th percentile	− 0.11
Deep learning (*n* = 4)	DL feature-45	10.44
	DL feature-115	− 56.23
	DL feature-121	− 8.59
	DL feature-207	− 1.82
Radiomics + Deep learning (n = 6)	Wavelet-HHL-skewness	− 0.13
	DL feature-45	13.29
	DL feature-59	− 5.94
	DL feature-115	− 74.34
	DL feature-121	− 14.88
	DL feature-125	1.79

*GLCM* Gray-level co-occurrence matrix, *LoG* Laplacian of Gaussian, *DL* Deep learning

### Performance of AI models

The performance metrics of the AI models and radiologists on the testing set are summarized in Table 4. The accuracy of models in descending order were ensemble model 82%, DL model 73%, clinical model 73%, combined radiomics and DL model 71%, and radiomics model 61%. The best performing model was the ensemble model with the highest accuracy (82%), sensitivity (68%), negative predictive rate (85%), and F1 score (0.72). The ensemble model achieved a specificity of 89%, AUC of 0.83, and positive predictive rate of 77%. The DL model had the highest AUC (0.89), specificity (100%), and positive predictive rate (100%) but the lowest sensitivity (21%).

**Table 4:** Performance metrics of AI models and radiologists

		Accuracy	Sensitivity	Specificity	AUC	Positive predictive rate	Negative predictive rate	F1 score
AI models	Radiomics	0.61	0.32	0.76	0.66	0.40	0.68	0.35
	DL	0.73	0.21	1	0.89	1	0.71	0.35
	Clinical	0.73	0.53	0.84	0.82	0.63	0.78	0.57
	Radiomics + DL	0.71	0.37	0.89	0.82	0.64	0.73	0.47
	Ensemble*	0.82	0.68	0.89	0.83	0.77	0.85	0.72
Radiologists without AI assistance	Radiologist 1	0.63	0.58	0.65	0.61	0.46	0.75	0.51
	Radiologist 2	0.64	0.58	0.68	0.63	0.48	0.76	0.52
	Radiologist 3	0.70	0.84	0.62	0.73	0.53	0.88	0.65
		Krippendorff’s alpha		0.4757				
	Radiologist 4	0.86	0.68	0.95	0.82	0.87	0.85	0.77
	Radiologist 5	0.79	0.95	0.70	0.83	0.62	0.96	0.75
		Krippendorff’s alpha		0.4806				
Radiologists with AI assistance	Radiologist 1	0.77	0.74	0.78	0.76	0.64	0.85	0.68
	Radiologist 2	0.80	0.89	0.76	0.83	0.65	0.93	0.76
	Radiologist 3	0.86	0.84	0.86	0.85	0.76	0.91	0.80
		Krippendorff’s alpha		0.6333				
	Radiologist 4	0.88	0.79	0.92	0.85	0.83	0.89	0.81
	Radiologist 5	0.82	0.84	0.81	0.83	0.70	0.91	0.76
		Krippendorff’s alpha		0.7331				

^*^Ensemble = radiomics + DL + clinical

Junior radiologists: radiologist 1–3

Senior radiologists: radiologist 4–5

*AI* Artificial intelligence, *AUC* Area under the ROC Curve, *DL* Deep learning

### Performance of radiologists

The senior radiologists achieved higher accuracy, specificity, AUC, positive predictive rate, and F1 score than all junior radiologists (Table 4). With AI model assistance, all junior radiologists showed an overall improvement in performance metrics, while the senior radiologists had only mild improvement in accuracy, AUC, and F1 score. The interobserver reliability of junior radiologists (Krippendorff’s alpha, 0.4757 vs 0.6333) and senior radiologists (Krippendorff’s alpha, 0.4806 vs 0.7331) also revealed improvement with AI assistance. The averaged performance results of radiologists are summarized in Table 5. With the assistance of ensemble model, the junior radiologists achieved a significant improvement in averaged accuracy (81% vs 66%), sensitivity (82% vs 67%), and specificity (80% vs 65%) that were comparable with senior radiologists. The senior radiologists only displayed a mild improvement in average accuracy (85% vs 83%) and specificity (87% vs 83%) and the same sensitivity (82%) with AI assistance. Aided by the ensemble-produced probabilities, junior radiologists also achieved an improvement in AUC that showed no statistically significant difference from senior radiologists. Comparisons of AUC between radiologists can be found in Additional file 1: Tables S5–S7.

**Table 5:** Performance comparison of radiologists and ensemble model

	Without AI	With AI	Ensemble model
*Junior radiologists averaged*			
Accuracy	0.66	0.81	0.82
Sensitivity	0.67	0.82	0.68
Specificity	0.65	0.80	0.89
*Senior radiologists averaged*			
Accuracy	0.83	0.85	0.82
Sensitivity	0.82	0.82	0.68
Specificity	0.83	0.87	0.89

*AI* artificial intelligence

### Performance comparison of ensemble model and radiologists

**Fig. 4:**
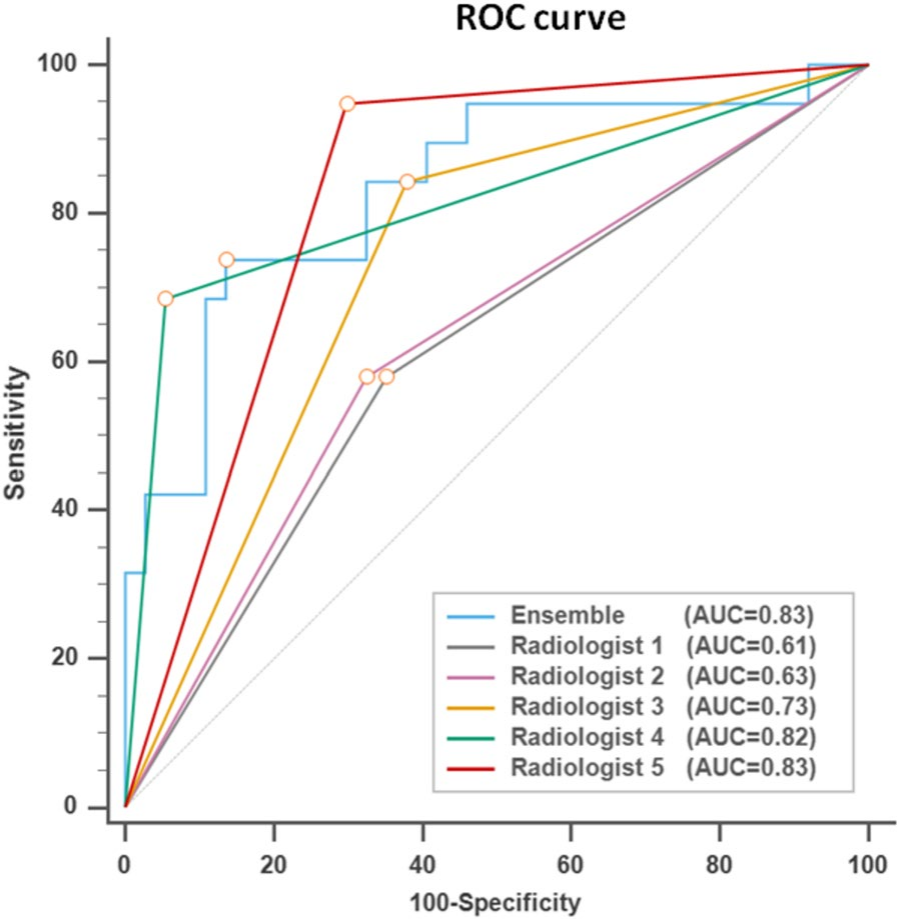
ROC curves of ensemble model and radiologists

Figure 4 demonstrates the ROC curves of ensemble model and radiologists. The AUC of ensemble model (0.83) was comparable with senior radiologists (0.82–0.83) and better than junior radiologists (0.61–0.73). Compared with junior radiologists averaged results (Table 5), the ensemble model had higher accuracy (82% vs 66%) and specificity (89% vs 65%) with comparable sensitivity (68% vs 67%). Against the senior radiologists averaged results, the ensemble model had a comparable accuracy (82% vs 83%), higher specificity (89% vs 83%), but lower sensitivity (68% vs 82%). Comparison of AUC between the ensemble model and radiologists can be found in Additional file 1: Table S8.

### Sample misclassified by AI model and/or radiologists

**Fig. 5:**
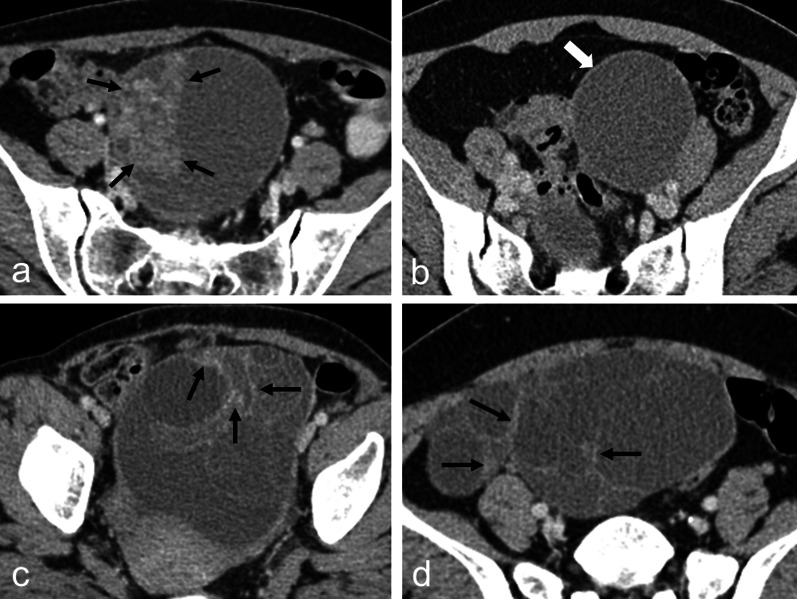
Contrast-enhanced CT images of ovarian tumors that were misclassified by AI model or/and junior radiologists. **a** A malignant ovarian tumor (clear cell carcinoma) that was predicted to be benign by AI model but malignant by all junior radiologists. The solid portion (arrow) in the tumor is a clue for malignancy in radiological evaluation. **b** A benign ovarian tumor (endometrioma) that was predicted to be malignant by AI model but benign by all junior radiologists. There was no solid portion, mural nodule, or thick septa to indicate malignancy in radiological evaluation. **c** A benign ovarian tumor (mucinous cystadenoma) that was predicted to be malignant by both AI model and all junior radiologists. **d** A benign ovarian tumor (mucinous cystadenoma) that was predicted to be malignant by all junior radiologists but benign by AI model. Thick septa (arrow) in **c** and **d** raised the suspicion of malignancy in radiological evaluation

Figure 5 demonstrates examples of tumor misclassified by AI model and/or radiologists under three scenarios. Figures 5a and b depict ovarian tumors that were misclassified by AI model but correctly differentiated by all junior radiologists, selected from 4 cases of this scenario, including 2 malignant and 2 benign tumors. Figure 5c demonstrated the only one tumor that was misclassified by both AI model and all junior radiologists. Figure 5d depicted an ovarian tumor that was wrongly differentiated by all 3 junior radiologists but correctly classified by AI model, selected from 9 cases of this scenario, including 1 malignant and 8 benign tumors.

## Discussion

In this study, we developed a CT-based AI model incorporating radiomics and DL features with clinical data to classify benign and malignant ovarian tumors using ML classifiers. The model can distinguish benign from malignant ovarian tumors with high accuracy (82%) and specificity (89%) for a fair sensitivity (68%). The model performed better than the junior radiologists’ average results. With the probabilities provided by the model, the junior radiologists showed a significant improvement in performance approaching to senior radiologists. These results demonstrate that the AI model can assist less-experienced radiologists in assessing ovarian tumors, providing evidence of the clinical validity of this model.

This is the first study applying ML combined with radiomics and DL features extracted from CT images to differentiate between benign and malignant ovarian tumors. There is limited research on applying DL to differentiate ovarian tumor using CT images. Christiansen et al. [29] and Wang et al. [30] applied DL for ovarian tumor differentiation using ultrasound and MRI respectively. Both studies used the CNN to build an end-to-end classification model which needed to be trained with a larger dataset. However, under common medical conditions, collecting a large uniform tumor image dataset with pathological diagnosis is very difficult. DL features, quantified image features extracted through an encoder-decoder CNN [31–34], may provide an alternative way for tumor imaging analysis on a relatively small dataset. Wang et al. [24] extracted DL features from CT images to predict tumor recurrence in high-grade serous ovarian cancer. Xia et al. [25] developed a CT-based scheme to classify ground-glass lung nodules by fusing radiomics and DL features. So far, there is no study using DL features or incorporating radiomics with DL features to differentiate ovarian tumors. Since radiomics, DL features, and clinical data represent different characteristics of tumor, we assume that an AI model integrating these features can accurately distinguish benign and malignant ovarian tumors. The better performance of the ensemble model verified our assumption that radiomics, DL features, and clinical data may provide complementary information on ovarian tumors and work better together in distinguishing benign from malignancy.

ML is often considered as a black box. In order to understand the decisions and mistakes that the AI model and radiologists made, we analyzed three scenarios of misclassified results. In the first scenario where the tumors were misclassified by AI model but correctly differentiated by all junior radiologists, the malignant tumor (Fig. 5a) had obvious solid portion, while the benign one (Fig. 5b) was a hypoattenuation tumor without solid portion or mural nodule. In traditional radiological evaluation, solid portion, mural nodule, and thick septa of an ovarian tumor are clues for malignancy. Tumors with typical CT image features, such as the above two tumors (Fig. 5a and b), would not be misdiagnosed by radiologists even though they were misclassified by AI model. In the second scenario where both AI model and all junior radiologists were wrong, the benign tumor (Fig. 5c) was a multiseptated cystic tumor with uneven thick septa that might raise the suspicion of malignancy in radiological evaluation. In the third scenario where the AI model was correct, but all junior radiologists were wrong, the tumor (Fig. 5d) was a benign multiseptated cystic tumor with thick septum. As mentioned before, it is challenging for radiologists in excluding the possibility of malignancy in such multiseptated cystic ovarian tumors. The AI model may do better than radiologists in identifying subtle features unexplainable by traditional radiological evaluation and help the radiologists to make correct decisions in difficult cases like the one in Fig. 5d.

The proposed model may potentially assist radiologists and gynecologists to assess ovarian tumors and guide therapeutic strategies for these patients, especially in hospitals that lack experienced radiologists. With the growing global physician shortage problem, the availability of an AI-assistance system is very important. Although MRI may provide better performance than CT in tumor differentiation due to its superior tissue contrast [36, 37], we believe a CT-based AI model would benefit more patients, especially those in remote areas. Although the sensitivity of our model is relatively low, its intended clinical application is not for screening. High specificity of the model is considerably more important than sensitivity since CT study usually serves as a confirmation modality for workup of indeterminate tumors on sonogram.

There are several limitations in this study. First, the data size is relatively small and without external validation cohort, and the study design is retrospective. Future studies using larger dataset from different institutions with prospective study design are essential to improve and validate the performance of the model. Second, manual segmentation of the ovarian tumors by a single radiologist can bias the results. However, considering accurate tumor segmentation is important for radiomics and DL feature extraction, we decided to use manual segmentation by an experienced radiologist. Third, recall of cases from the first session may be a concern when the radiologists were asked to reevaluate the CT images with AI assistance. To address this issue, we arranged a time delay of at least one month between the two sessions. Fourth, we chose CT as our imaging tool because it is far more available than MR. However, this remains a potential weakness for the developed tool applicability since an MRI-based model might outperform the proposed CT-based model. Fifth, we applied ML classifiers rather than DL method for tumor classification due to the limitation of small data size.

## Conclusions

In this study, we developed a CT-based AI model incorporating radiomics and DL features with clinical data to distinguish benign from malignant ovarian tumors using ML classifiers. The model can distinguish benign from malignant ovarian tumors with high accuracy and specificity. Besides, the model can improve the performance of less-experienced radiologists in assessing ovarian tumors, and potentially guide gynecologists to provide better therapeutic strategies for these patients.

## Supplementary Information

**Additional file 1. Table S1.** Radiomics features extracted in this study. **Table S2.** Patient and tumor characteristics for the training and testing sets. **Table S3.** Performance metrics of AI models on training set. **Table S4.** Performancemetrics of AI models on testing set. **Table S5.** Comparison of AUC between radiologists with and without AI assistance. **Table S6.** Comparison of AUC between junior radiologists and senior radiologists. **Table S7.** Comparison of AUC between junior radiologists with AI and senior radiologists. **Table S8.** Comparison of AUC between ensemble model and radiologists.

## Data Availability

The datasets used and/or analyzed during the current study are available from the corresponding author on reasonable request.

## Abbreviations

AI: Artificial intelligence
AUC: Area under the ROC curve
CA-125: Cancer antigen 125
CNN: Convolutional neural network
CT: Computed tomography
DL: Deep learning
GLCM: Gray-level co-occurrence matrix
KNN: K-nearest neighbor
LASSO: Least absolute shrinkage and selection operator
LoG: Laplacian of Gaussian
LR: Logistic regression
ML: Machine learning
MRI: Magnetic resonance imaging
RF: Random forest
RMSE: Root mean squared error
ROC: Receiver operating characteristic curve
SVM: Support vector machine
