# Supplementary material for: Machine learning combined with radiomics and deep learning features extracted from CT images: a novel AI model to distinguish benign from malignant ovarian tumors
Source: Insights Imaging. 2023 Apr 24;14:68. doi: 10.1186/s13244-023-01412-x (PMC10126170; doi:10.1186/s13244-023-01412-x)
Supplement: Supplementary file 1 — Additional file 1. Table S1. Radiomics features extracted in this study. Table S2. Patient and tumor characteristics for the training and testing sets. Table S3. Performance metrics of AI models on training set. Table S4. Performancemetrics of AI models on testing set. Table S5. Comparison of AUC between radiologists with and without AI assistance. Table S6. Comparison of AUC between junior radiologists and senior radiologists. Table S7. Comparison of AUC between junior radiologists with AI and senior radiologists. Table S8. Comparison of AUC between ensemble model and radiologists. [file 13244_2023_1412_MOESM1_ESM.pdf]

Electronic Supplementary Material

Machine learning combined with radiomics and deep learning features extracted from CT images: A novel AI model to distinguish benign from malignant ovarian tumors

Supplementary Table 1. Radiomics features extracted in this study.

Histogram (n=12)	LoG (n=12)	Wavelet (n=96)	GLCM (n=9)
Mean	Mean	Mean (LLL, LLH, LHL, LHH, HLL, HLH, HHL, HHH)	Homogeneity
Standard deviation	Standard deviation	Standard deviation (LLL, LLH, LHL, LHH, HLL, HLH, HHL, HHH)	Energy
Entropy	Entropy	Entropy (LLL, LLH, LHL, LHH, HLL, HLH, HHL, HHH)	Contrast
5th percentile	5th percentile	5th percentile (LLL, LLH, LHL, LHH, HLL, HLH, HHL, HHH)	Dissimilarity
10th percentile	10th percentile	10th percentile (LLL, LLH, LHL, LHH, HLL, HLH, HHL, HHH)	Autocorrelation
25th percentile	25th percentile	25th percentile (LLL, LLH, LHL, LHH, HLL, HLH, HHL, HHH)	Entropy
50th percentile	50th percentile	50th percentile (LLL, LLH, LHL, LHH, HLL, HLH, HHL, HHH)	Sum average
75th percentile	75th percentile	75th percentile (LLL, LLH, LHL, LHH, HLL, HLH, HHL, HHH)	Correlation
90th percentile	90th percentile	90th percentile (LLL, LLH, LHL, LHH, HLL, HLH, HHL, HHH)	Variance
95th percentile	95th percentile	95th percentile (LLL, LLH, LHL, LHH, HLL, HLH, HHL, HHH)	
Skewness	Skewness	Skewness (LLL, LLH, LHL, LHH, HLL, HLH, HHL, HHH)	
Kurtosis	Kurtosis	Kurtosis (LLL, LLH, LHL, LHH, HLL, HLH, HHL, HHH)	

Notes: LoG, Laplacian of Gaussian; GLCM, gray-level co-occurrence matrix.

Supplementary Table 2. Patients and tumor characteristics for the training and testing sets.

	**Training set (n=129)**	**Testing set (n=56)**	***p* value**
**Benign**	75 (58.1%)	37 (66.1%)	0.3118
**Malignant**	54 (41.9%)	19 (33.9%)	
**Age (years)**	47.1 ± 14.1	45.0 ± 15.3	0.3696
**Volume (cm^3)**	732.9 ± 1101.8	551.6 ± 785.7	0.2667
**CA-125**			
≤ 35 U/mL	61 (47.3%)	23 (41.1%)	0.4366
> 35 U/mL	68 (52.7%)	33 (58.9%)	
**Side**			
Unilateral	89 (69.0%)	38 (67.9%)	0.8788
Bilateral	40 (31.0%)	18 (32.1%)	

Notes: All values are expressed as the mean ± SD or number (%); CA-125, cancer antigen 125.

Electronic Supplementary Material

Supplementary Table 3. Performance metrics of AI models on training set.

	Accuracy	Sensitivity	Specificity	AUC	Positive Predictive Rate	Negative Predictive Rate	F1 Score
**Radiomics**							
KNN	0.59	0.59	0.59	0.65	0.51	0.67	0.55
SVM	0.61	0.06	1	0.70	1	0.60	0.11
LR	0.61	0.41	0.76	0.71	0.55	0.64	0.47
RF	0.58	0.48	0.65	0.71	0.50	0.64	0.51
**DL**							
KNN	0.61	0.56	0.65	0.61	0.54	0.67	0.55
SVM	0.58	0	1	0.58	-	0.58	-
LR	0.65	0.28	0.92	0.64	0.71	0.64	0.40
RF	0.74	0.67	0.80	0.82	0.70	0.76	0.67
**Clinical**							
KNN	0.66	0.58	0.70	0.65	0.60	0.71	0.59
SVM	0.56	0.61	0.52	0.66	0.48	0.65	0.54
LR	0.75	0.67	0.81	0.77	0.72	0.77	0.69
RF	0.76	0.65	0.84	0.76	0.75	0.77	0.69
**Radiomics + DL**							
KNN	0.71	0.69	0.72	0.71	0.64	0.76	0.66
SVM	0.58	0	1	0.73	-	0.58	-
LR	0.73	0.52	0.88	0.80	0.76	0.72	0.62
RF	0.78	0.70	0.83	0.85	0.75	0.80	0.72
**Ensemble**							
KNN	0.68	0.59	0.75	0.67	0.63	0.72	0.61
SVM	0.68	0.50	0.81	0.72	0.66	0.69	0.57
LR	0.77	0.65	0.85	0.80	0.76	0.77	0.70
RF	0.80	0.74	0.84	0.86	0.77	0.82	0.75

Notes: AI, artificial intelligence; AUC, area under the ROC curve; DL, deep learning; KNN, K-nearest neighbor; SVM, support vector machine; LR, logistic regression; RF, random forest.

Electronic Supplementary Material

Supplementary Table 4. Performance metrics of AI models on testing set.

	Accuracy	Sensitivity	Specificity	AUC	Positive Predictive Rate	Negative Predictive Rate	F1 Score
**Radiomics**							
KNN	0.59	0.47	0.65	0.56	0.41	0.71	0.44
SVM	0.66	0	1	0.59	-	0.66	-
LR	0.61	0.32	0.76	0.66	0.40	0.68	0.35
RF	0.70	0.53	0.78	0.75	0.56	0.76	0.54
**DL**							
KNN	0.66	0.53	0.73	0.63	0.50	0.75	0.51
SVM	0.66	0	1	0.68	-	0.66	-
LR	0.73	0.21	1	0.89	1	0.71	0.35
RF	0.68	0.47	0.78	0.70	0.53	0.74	0.50
**Clinical**							
KNN	0.68	0.45	0.75	0.60	0.53	0.72	0.47
SVM	0.71	0.26	0.95	0.79	0.71	0.71	0.38
LR	0.73	0.53	0.84	0.82	0.63	0.78	0.57
RF	0.75	0.47	0.89	0.73	0.69	0.77	0.56
**Radiomics + DL**							
KNN	0.71	0.53	0.81	0.67	0.59	0.77	0.56
SVM	0.66	0	1	0.57	-	0.66	-
LR	0.71	0.37	0.89	0.82	0.64	0.73	0.47
RF	0.68	0.53	0.76	0.75	0.53	0.76	0.53
**Ensemble**							
KNN	0.70	0.47	0.81	0.64	0.56	0.75	0.51
SVM	0.36	0.74	0.16	0.50	0.31	0.55	0.44
LR	0.82	0.68	0.89	0.83	0.77	0.85	0.72
RF	0.75	0.58	0.84	0.83	0.65	0.80	0.61

Notes: AI, artificial intelligence; AUC, area under the ROC curve; DL, deep learning; KNN, K-nearest neighbor; SVM, support vector machine; LR, logistic regression; RF, random forest.

Electronic Supplementary Material

Supplementary Table 5. Comparison of AUC between radiologists with and without AI assistance.

	AUC		|Δ|	95% CI	p-value
	Without AI	With AI			
**Radiologist 1**	0.61 (0.47-0.74)	0.76 (0.63-0.86)	0.15	(0.010, 0.283)	0.0358
**Radiologist 2**	0.63 (0.49-0.75)	0.83 (0.70-0.91)	0.20	(0.076, 0.321)	0.0015
**Radiologist 3**	0.73 (0.60-0.84)	0.85 (0.73-0.93)	0.12	(-0.005, 0.249)	0.0606
**Radiologist 4**	0.82 (0.69-0.91)	0.85 (0.73-0.93)	0.03	(-0.037, 0.115)	0.3110
**Radiologist 5**	0.83 (0.70-0.91)	0.83 (0.70-0.92)	0	(-0.094, 0.097)	0.9766

Junior radiologists: radiologist 1-3.
Senior radiologists: radiologist 4-5.
Notes: AI, artificial intelligence; AUC, area under the ROC curve.

Supplementary Table 6. Comparison of AUC between junior radiologists and senior radiologists.

	AUC			|Δ|	95% CI	p-value
	Junior radiologist	Senior radiologist				
**Radiologist 1**	0.61 (0.47-0.74)	**Radiologist 4**	0.82 (0.69-0.91)	0.21	(0.036, 0.367)	0.0173
		**Radiologist 5**	0.83 (0.70-0.91)	0.22	(0.067, 0.356)	0.0041
**Radiologist 2**	0.63 (0.49-0.75)	**Radiologist 4**	0.82 (0.69-0.91)	0.19	(0.062, 0.314)	0.0035
		**Radiologist 5**	0.83 (0.70-0.91)	0.20	(0.071, 0.324)	0.0022
**Radiologist 3**	0.73 (0.60-0.84)	**Radiologist 4**	0.82 (0.69-0.91)	0.09	(-0.072, 0.239)	0.2942
		**Radiologist 5**	0.83 (0.70-0.91)	0.20	(0.071, 0.324)	0.0022

Junior radiologists: radiologist 1-3.
Senior radiologists: radiologist 4-5.
Notes: AUC, area under the ROC curve.

Supplementary Table 7. Comparison of AUC between junior radiologists with AI and senior radiologists.

	AUC			|Δ|	95% CI	p-value
	Junior radiologist with AI	Senior radiologist				
**Radiologist 1**	0.76 (0.63-0.86)	**Radiologist 4**	0.82 (0.69-0.91)	0.06	(-0.083, 0.192)	0.4357
		**Radiologist 5**	0.83 (0.70-0.91)	0.07	(-0.080, 0.209)	0.3791
**Radiologist 2**	0.83 (0.70-0.91)	**Radiologist 4**	0.82 (0.69-0.91)	0.01	(-0.126, 0.147)	0.8781
		**Radiologist 5**	0.83 (0.70-0.91)	0.07	(-0.080, 0.209)	0.3791
**Radiologist 3**	0.85 (0.73-0.93)	**Radiologist 4**	0.82 (0.69-0.91)	0.03	(-0.057, 0.134)	0.4296
		**Radiologist 5**	0.83 (0.70-0.91)	0.02	(-0.065, 0.121)	0.5488

Junior radiologists: radiologist 1-3.
Senior radiologists: radiologist 4-5.
Notes: AI, artificial intelligence; AUC, area under the ROC curve.

Electronic Supplementary Material

Supplementary Table 8. Comparison of AUC between ensemble model and radiologists.

	AUC		|Δ|	95% CI	p-value
	**Radiologist**	**Ensemble**			
**Radiologist 1**	0.61 (0.47-0.74)	0.83 (0.71-0.92)	0.22	(0.078, 0.362)	0.0025
**Radiologist 2**	0.63 (0.49-0.75)		0.20	(0.011, 0.402)	0.0386
**Radiologist 3**	0.73 (0.60-0.84)		0.10	(-0.068, 0.272)	0.2409
**Radiologist 4**	0.82 (0.69-0.91)		0.01	(-0.139, 0.176)	0.8179
**Radiologist 5**	0.83 (0.70-0.91)		0	(-0.142, 0.159)	0.9115

Junior radiologists: radiologist 1-3.
Senior radiologists: radiologist 4-5.
Notes: AUC, area under the ROC curve.
